# Challenges and Practical Solutions to MRI and Histology Matching and Measurements Using Available ImageJ Software Tools

**DOI:** 10.3390/biomedicines10071556

**Published:** 2022-06-30

**Authors:** Marina Y. Khodanovich, Tatyana V. Anan’ina, Elena P. Krutenkova, Andrey E. Akulov, Marina S. Kudabaeva, Mikhail V. Svetlik, Yana A. Tumentceva, Maria M. Shadrina, Anna V. Naumova

**Affiliations:** 1 Laboratory of Neurobiology, Research Institute of Biology and Biophysics, Tomsk State University, Russia. 36, Lenina Ave., 634050 Tomsk, Russia; tany_a@list.ru (T.V.A.); len--k@yandex.ru (E.P.K.); kmsra08@gmail.com (M.S.K.); mihasv@gmail.com (M.V.S.); mimizyana@gmail.com (Y.A.T.); m.m.shadrina@list.ru (M.M.S.); nav@uw.edu (A.V.N.); 2 Institute of Cytology and Genetics, The Siberian Branch of the Russian Academy of Sciences, 10 Lavrentyeva Avenue, 630090 Novosibirsk, Russia; akulov.mri@gmail.com; 3 Department of Radiology, University of Washington, 850 Republican Street, Seattle, WA 98109, USA

**Keywords:** magnetic resonance imaging, MRI, histology, immunofluorescence, quantification, registration, ImageJ, brain

## Abstract

Traditionally histology is the gold standard for the validation of imaging experiments. Matching imaging slices and histological sections and the precise outlining of corresponding tissue structures are difficult. Challenges are based on differences in imaging and histological slice thickness as well as tissue shrinkage and alterations after processing. Here we describe step-by-step instructions that might be used as a universal pathway to overlay MRI and histological images and for a correlation of measurements between imaging modalities. The free available (Fiji is just) ImageJ software tools were used for regions of interest transformation (ROIT) and alignment using a rat brain MRI as an example. The developed ROIT procedure was compared to a manual delineation of rat brain structures. The ROIT plugin was developed for ImageJ to enable an automatization of the image processing and structural analysis of the rodent brain.

## 1. Introduction

Magnetic resonance imaging (MRI) provides important information about anatomy and pathology, allowing a non-invasive assessment of an organ’s structure and function. Novel MRI methods have been developed for the evaluation of tissue composition (e.g., conducting tracts, myelin, collagen) or specific pathological conditions (ischemia, demyelination, inflammation). Many of these methods are positioned as quantitative; therefore, they must be histologically validated in experimental animal studies to provide the foundation for further clinical applications. Quantitative MRI techniques with an improved specificity to myelin have been rapidly developed in recent decades, such as methods based on single- or multi-component relaxation [1,2,3], magnetization transfer [4,5,6,7,8,9], anisotropic diffusion [10], and magnetic susceptibility [11,12]. The gold standards for confirming the accuracy of MRI myelin estimates are Luxol Fast Blue (LFB) histology staining, immunohistochemistry for myelin basic protein (MBP), or proteolipid protein (PLP) [13]. Histological validation of MRI findings is an important component of animal models of cerebrovascular [14] and neurodegenerative [15] pathologies, animal tumor models [16], human post-MRI studies when the treatment includes resections [17,18,19,20], and human post-MRI post-mortem histopathology [21]. Cell transplantation studies [22,23,24,25,26,27,28,29,30,31,32] and gene reporter imaging [33,34,35] also require histological validation.

Typically, the validation of new MRI techniques includes an evaluation of the relationship between MRI and histological measurements in anatomically similar areas or regions of interest (ROI). The reliability of the correlations between in vivo and ex vivo data is determined by the similar anatomy in the ROIs on MRI and histology images. First, MRI and histological sections should have the same spatial location, which is determined by the coordinate perpendicular to the slice plane (for example, along the anteroposterior axis for coronal slices), as well as the slope of the slice relative to this axis (sagittal plane in the case of coronal slices). The slope of histological sections cannot be changed once they have been obtained. However, for 3D MRI images, the slope of slice can be corrected by free rotation (linear interpolation) of the slice plane, implemented, for example, in MRIcron or ImageJ free software.

A more serious problem is that postmortem sections undergo distortion during tissue processing which may include shrinkage, tears, and folds. For this reason, the identification of anatomically matching ROIs on MR images and histological sections is crucial. Several methods are used to solve this problem. The first approach includes the manual segmentation of both types of images based on a visual correspondence of histology images with MRI slices [7,8,9,35]. This approach is entirely based on the qualifications of the operator and his subjective assessment. Additional limitations of this approach are the difficulty of transferring the results of automatic segmentation of MRI to histology images, as well as difficulties in tissue segmentation when the ROI boundaries are not obvious (for example, the boundaries of inflammation within an ischemic lesion). Another approach is the co-registration of histological slices to corresponding in vivo MRI slices by elastic (non-rigid) transformations of the histology image. The main limitation of this approach is the deformation of the image, which may result in an incorrect cell calculation and other parameters in the ROIs [16,36].

We propose here a new simple approach based on an adequate transformation of ROIs for the explored images while the original raw MR and histological images remain unchanged. Free available Fiji (ImageJ) software [37] was employed for the development of the method and its validation using a quantitative comparison of ROIs from rodent brain MR images and corresponding histology sections of the same brain.

The developed method of ROI transformation (ROIT) can be applied to solve the problems of matching measurements between MRI and histology images. The ROIT method can be used for MRI-to-histology enlargement/transformation of ROI as well as for histology-to-MRI ROI’s diminishment approach. The first approach of ROI enlargement with transformation assumes that the ROI can be easily identified on MRI and then transferred to the histological image for appropriate measurements, for example, for the quantification of the myelin content. On the contrary, the second approach, ROI diminishment with transformation, involves a quantitative assessment of changes in the MRI signal, which are caused by histologically identifiable biological features. An example of the second approach is the quantification of the MR signal after the injection of paramagnetic nanoparticles and gene reporters.

## 2. Materials and Methods

### 2.1. Dataset Description

Two datasets were chosen to validate the proposed method of smart transformation of the regions of interest (ROIs). The first dataset (MPF-LFB dataset) included MR images obtained by macromolecular proton fraction (MPF) mapping [8,38,39] and the transmitted light micrographs of brain slices stained for myelin with LFB. MPF is a biophysical parameter that shows a strong correlation with myelin content in a large number of MR-histology studies on different animal models including the normal rat brain [40], cuprizone-induced demyelination [7] and remyelination [9], and local brain ischemia [8]. MPF maps were reconstructed from three spoiled gradient-echo sequences providing MT-, T1-, and proton-density-weighted images and using a single-point synthetic reference method [8,38,39]. Software for MPF map reconstruction (https://www.macromolecularmri.org/, accessed on 1 June 2022) was provided by the National Institutes of Health (NIH) High-Impact Neuroscience Research Resource “Quantitative myelin mapping in vivo for clinical and pre-clinical MRI” (NIH grant R24NS104098).

The second dataset (T2-DAPI dataset) included T2-weighted images and the micrographs of brain cryosections in which cell nuclei were stained with DAPI (4,6-diamidino-2-phenylindole). DAPI was used for validation, as it is present in almost any fluorescent staining as part of the mounting medium.

Both datasets (n = 10 in each dataset) included in vivo MRI and histological data of intact male Sprague-Dawley rats (weight 250–270 g). Animals were bred and housed in the specific pathogen-free (SPF) vivarium of the Center for Genetic Resources of Laboratory Animals at the Institute of Cytology and Genetics of the Siberian Branch of the Russian Academy of Sciences (ICG SB RAS) in individually ventilated cages (one animal per cage, 10/14 light/dark cycle, temperature of 22 to 24 °C, humidity of 40 to 50%, water and granulated chow ad libitum). All procedures were approved by the Bioethical Committees at Tomsk State University and ICG SB RAS (MPF-LFB dataset: protocol #25 dated 12 December 2014, T2-DAPI dataset: protocol #26 dated 20 March 2019). Six out of ten images in the MPF-LFB dataset and five out of ten images in the T2-DAPI dataset were obtained from animals that underwent brain ischemia using the middle cerebral artery (MCAO) occlusion model by Longa’s method in which a filament was inserted into the circle of Willis through the external carotid artery to occlude the middle cerebral artery for 1 h followed by reperfusion [8,33]. MRI data were acquired on an 11.7T Biospec small-animal MRI scanner (Bruker Biospin, MRI GmbH, Ettlingen, Germany). During the MRI, the animals were anesthetized with isoflurane (1.5–2% in combination with 100% oxygen). The animals after MCAO were scanned at the different time points (1, 3, 10, and 30 days after MCAO). After MRI, rats were transcardially perfused with 4% paraformaldehyde in phosphate buffer solution (PBS), brains were removed, cryoprotected (sucrose in PBS, 24 h at 10% and 24 h at 20%, 4 °C), frozen in liquid nitrogen vapor, and stored at −80 °C for further histological or immunochemical processing. The histology data consisted of the micrographs of 10 µm brain cryosections obtained from the same animal at the brain location from −1.92 mm to +1.74 mm from bregma, according to a rat brain atlas [40].

The MPF-LFB dataset included whole-brain 3D MPF maps (field of view 36 × 36 mm^2^, acquisition matrix 180 × 180 × 90, final resolution 0.2 × 0.2 × 0.4 mm^3^) and histological micrographs of brain slices stained for myelin with LFB. Whole-brain sections were photographed using an Axio Imager Z2 microscope (Carl Zeiss, Germany) with AxioVision 4.8 software using 1× objective. Identical imaging parameters (voltage of a microscope lamp and exposure time) were set for all photographed slices.

MRI data of the T2-DAPI dataset included T2-weighted images (field of view 36 × 36 mm^2^, acquisition matrix 300 × 300 × 12, final resolution 0.12 × 0.12 × 1.5 mm^3^) and the fluorescent micrographs of brain cryosections stained immunohistochemically for ferritin and embedded in VECTASHIELD mounting medium (Vector Laboratories, Burlingame, CA, USA) with DAPI. Fluorescent microscopy was performed using an Axio Imager Z2 microscope (Carl Zeiss, Oberkochen, Germany) with 10× objective and AxioVision 4.8 (Carl Zeiss, Oberkochen, Germany) software with a MozaiX module, which enables the creation of whole brain images by means of stitching together smaller images. Identical imaging parameters were set for all photographed sections. The blue channel represented DAPI fluorescence used for method validation.

### 2.2. Image Processing

Image processing was performed using the free available software (Fiji is just) ImageJ 1.53f51 (National Institutes of Health, Bethesda, MD, USA) [37,41]. To validate the developed method of ROI transformation (ROIT), which is described below, the corpus callosum (CC) was chosen as the brain structure that can be easily identified in most MR and histological images of the brain excluding the most rostral and caudal slices. Image processing for method validation according to the ROI transformation protocol was performed by two qualified operators with PhD degrees and knowledge of rodent brain structures. Each operator manually delineated two ROIs (source ROIs) on matched histology and MR images: (1) CC, (2) outline of a whole brain slice.

The two operators performed image processing for both datasets. Each operator applied the ROIT method to transform ROIs from MR to histology images and from histology images to MR. Additionally, one operator manually placed circular ROIs of the standard size in the center of the ischemic lesion of MPF maps and anatomically matched the LFB stained images. The lesion was defined by hypointensity on MPF maps and less intense LFB staining on histological sections. A similar ROI was placed at the symmetrical anatomic location in the contralateral hemisphere. The ROI size was selected to be completely within the ischemic lesion. For sham-operated animals, ROIs were placed approximately in the center of the caudoputamen. LFB optical density (OD) was quantified from the intensity of the red channel on RGB images with background correction as previously described [5,7,8].

### 2.3. Statistical Analysis

All statistical analyses were performed using Statistica 10.0 (StatSoft, Inc., Tusla, OK, USA) software. To quantitatively assess the similarity between the source and transformed ROIs, as well as between the manual outlining of the two operators, the Dice similarity coefficient (DC) was calculated. The values of DC for CC and the brain section were analyzed using a repeated measures ANOVA with the Greenhouse–Geisser correction followed by Bonferroni post-hoc tests for multiple comparisons. The correspondence between the measurements made by the traditional method of manual ROI placement and ROIT method was also assessed using repeated measures ANOVA with the Greenhouse–Geisser correction followed by Bonferroni post-hoc tests for multiple comparisons. To assess the relationships between MPF and LFB OD, percentage changes in LFB OD and MPF were assessed using the Pearson correlation coefficient (r). Statistical significance was defined as a *p* value less than 0.05.

### 2.4. ROI Transformation Protocol (ROIT Method)

**Figure 1:**
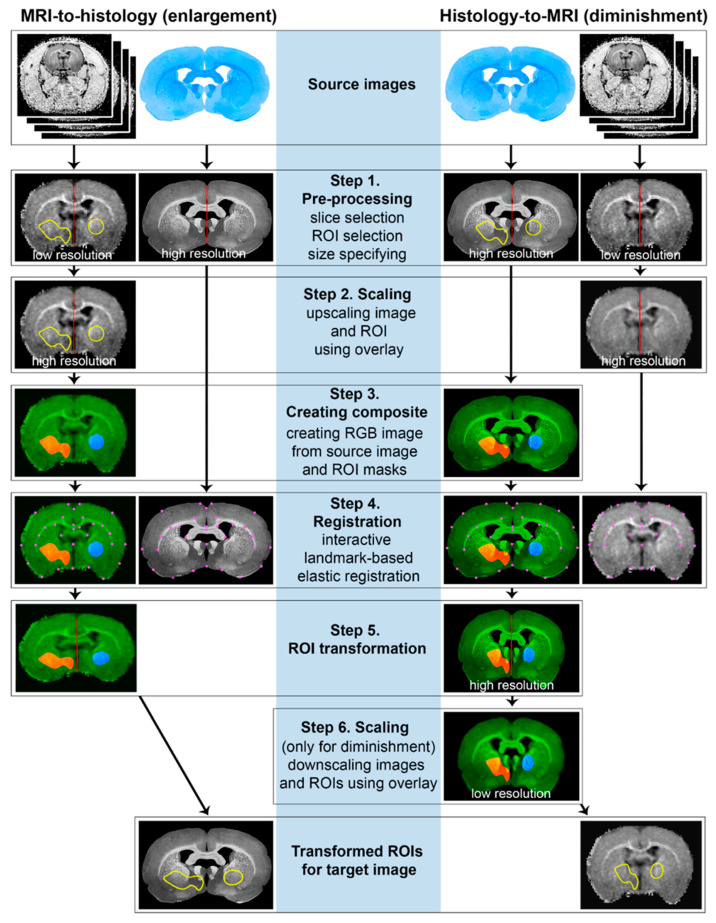
Step-by-step schematic diagram of the ROIT method algorithm for “MRI-to-histology” and “Histology-to-MRI” transformations.

Figure 1 shows a step-by-step schematic diagram of the algorithm of the ROIT method for “MRI-to-histology” and “Histology-to-MRI” transformations. Algorithm steps included source image pre-processing and region definition (Step 1), MR image scaling to higher resolution (Step 2), RGB composite image generation (Step 3), registration (Step 4), obtaining of co-registered composite image with transformed ROIs as a color channel (Step 5), scaling the histology image to lower resolution (Step 6) and obtaining the final ROIs by decomposing the composite image into channels. Steps 2–6 were combined into a plugin “ROIT” for processing automation, provided as Supplementary Jar S1 and available online (https://drive.google.com/file/d/1BE_S4zaslwfA4nzgx5xjhITbQrWU20Gs/view, accessed on 26 May 2022), which can be installed in ImageJ and used to apply the ROIT method. Additionally, a video tutorial of the ROIT method application is provided as Supplementary Video S1.

*Step 1. Pre-processing*

**Figure 2:**
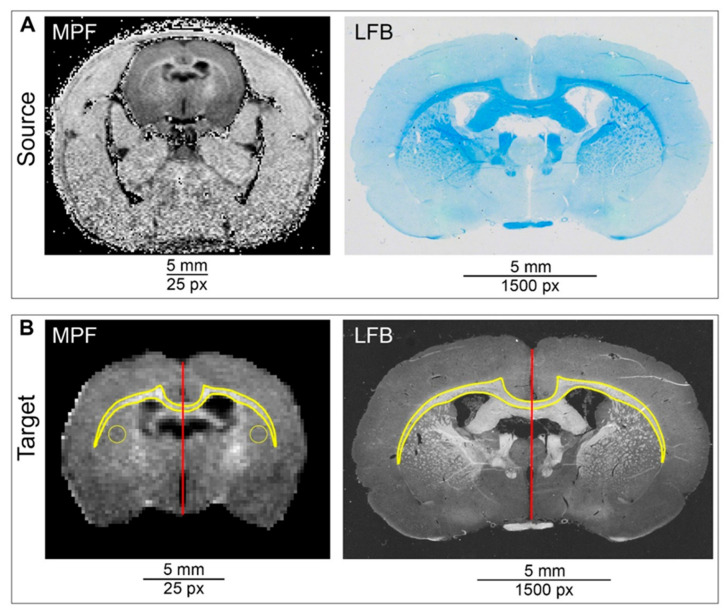
Pre-processing of MR and histological images. (**A**) Example of source (MR) and target (histological) images. Source image is represented by one slice of 3D MPF map; target image is presented by a micrograph of anatomically matched brain section stained with LFB. (**B**) Source and target images after pre-processing with ROI selection. ROIs for measurements and method validation (delineated corpus callosum and two symmetrical round ROIs in the left and right caudoputamen) are marked with a yellow line. Linear red line ROIs are added for a vertical size assessment of the source and target images which are necessary for further transformation and scaling.

Two images of different sizes and resolution should be processed and matched: a low-resolution MR image and a high-resolution histological image (Figure 2).

The initial stage of processing includes the selection of the MR and histology slices that most closely match each other in the sagittal coordinate (Figure 2A). Pre-processing of the MR image includes the isolation of the selected 2D slice from the stack and image cropping (leaving the brain but removing skull bones and muscles from the image). These steps are optional but make the image registration easier in the next steps.

When a histology image is used as a source for the ROI transformation, pre-processing means the histology image transformation to grayscale. For transmitted light micrographs, the RGB image can be split into channels and then the most contrasted channel can be used (in the case of LBF staining, we used the red channel) or the main image can be converted to grayscale. We also inverted the red channel image (although this step is optional) to make it easier to compare MR and histological images during registration. The result of the pre-processing is shown in Figure 2B.

Immunofluorescent labeled multichannel micrographs can also be split into channels, then the image with the most clearly distinguishable brain structures can be used, for example, channels stained for axons and neurons. However, if none of the labels clearly identify brain structures, the stained cell nuclei for DAPI can be used.

In addition, it is necessary to designate regions of interest (ROIs) for measurements and transformation. ROIs should be manually delineated on the source image (MR or histological image, depending on the direction of transformation) using ImageJ selection tools (polygon, rectangle, circle or freehand) and added to the ROI manager. If it is necessary to obtain a curved selection, as in the case of the corpus callosum, the “Edit→Select→Fit Spline” command can be used. Since three color channels will be used to transform ROIs, and one of the channels will be the source image, only two channels can be used for transformation. If more regions are needed for transformation, several regions can be combined into one using the command “ROI manager→More→OR (Combine)”. Once the regions have been determined, appropriate measurements can be made for the source image. For further transformation, it is also necessary to specify the vertical linear size of the slice both on source and target images using the “Straight line” selection tool and add lines to ROI manager (Figure 2).

*Step 2. Upscaling of low-resolution image together with ROI as overlay*

Example ROIs (the delineated by polygon tool corpus callosum and combined ROI in the caudoputamen) are shown in Figure 2B.

The next important step is correctly enlarging the ROI selected on the MR image into a high-resolution histological image (or reducing if ROI transforms from histological to MR image). Although the ROIs in ImageJ are essentially vector graphics, the tools available do not allow you to directly enlarge the ROI along with the image. For the correct scaling, the ROIT method uses the ImageJ overlay tool. Overlays are non-active selections displayed over the pixel data, which, similar to active selections, are vector graphics. For further scaling, previously created ROIs (“straight lines”), corresponding to vertical slice dimensions on the MR and histology image, are used. Using overlays that match the ROIs allows you to keep the curved shape without “stepped” artifacts. Both the MRI-to-histology and histology-to-MRI transformation involve the scaling of MRI image to a high resolution, because this allows a registration with a higher quality. Scaling of the histological image with a decrease in resolution is performed after registration, at step 5.

*Step 3. Creating a composite image for registration*

**Figure 3:**
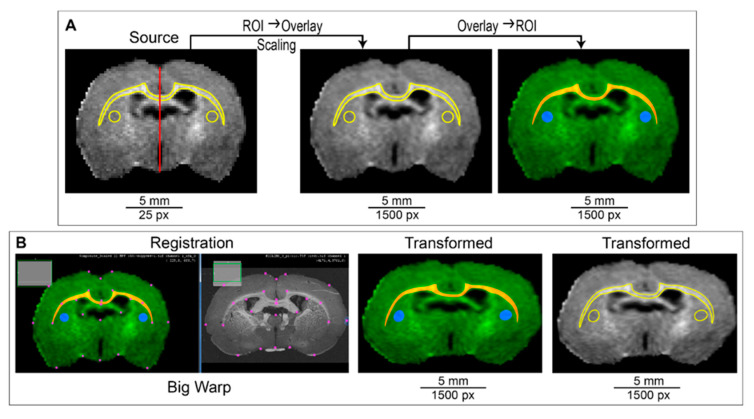
Steps 2–6 of the ROIT method. (**A**) Scaling of source image (step 2) and the creation of the RGB composite image (step 3). (**B**) Registration of the source MR image to target the histological image using the Big Warp tool and splitting a registered composite image into RGB channels to obtain transformed ROIs.

The overlay is not editable, except for scaling. It needs to be converted to an editable image, which would be registered together with the source image. To complete this, after scaling, the overlay is converted into ROIs. Each ROI is converted into a mask; all masks and the scaled source image are combined into a three-channel RGB image. The result is shown in Figure 3A.

*Step 4. Registration of source image to target image*.

The next step is an interactive landmark-based deformable registration of the source image to the target image. To complete this, various tools provided by Fiji (ImageJ) can be used, such as Big Warp [42], Landmark Correspondences [43], BUnwarpJ [44], etc. The developed plugin “ROIT” (Supplementary Jar S1) uses the Big Warp tool (Figure 3B). Landmarks for registration are selected manually. It is important that the registration is performed by a qualified operator who has a good knowledge of the anatomy of the brain. The most clearly defined points, such as intersections (e.g., intersection of the corpus callosum with the midline), corner apices, and intact slice edges, should be used as landmarks. We used no more than 30 landmarks (20 on average) for registration.

*Step 5. ROI transformation*

In the Big Warp plugin, the export of a composite image (moving image), co-registered to the target image (fixed image), is carried out using the “File→Export moving image” command. The plugin allows the user to return to registration if he is not satisfied with the result. The registration result is a RGB composite image, which is decomposed into color channels, which are converted into masks.

*Step 6. Downscaling of the high-resolution histological image together with ROI as an overlay (only for Histology-to-MRI transformation)*

The last step is different for MRI-to-histology and histology-to-MRI transformation. For the MRI-to-histology transformation, it only includes the conversion of binary masks into ROIs. For histology-to-MRI transformation, the co-registered to MRI histological image should be previously scaled in accordance with the low-resolution MRI image. For this, masks covert to overlays to avoid distortion, and after scaling they convert into ROIs. The ROIs obtained can be used for measurements.

## 3. Results

### 3.1. Correspondence of Manual Delineation between Two Operators

**Figure 4:**
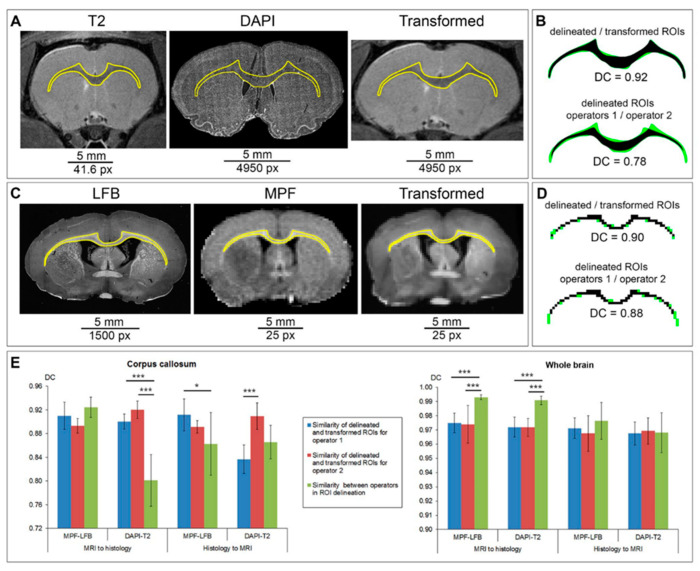
Correspondence between two operators in the manual delineation of the CC and brain section. (**A**) Examples of source MR and histology images from the LFB-MPF and DAPI-T2 datasets for manual delineation. The LFB image is presented as an inverted red channel of the source image. The grayscale DAPI image includes a magnified view of the delineated CC. (**B**) Correspondence of CC ROIs delineated in source images (**A**) by two operators with DC: black color represents matched pixels; green color represents mismatched pixels. (**C**–**E**) DCs of manual delineation of CC and brain section by two operators: dependence on the presence of an ischemic lesion (**C**), type of source image (**D**), and type of delineated structure (**E**). Error bars correspond to the SD. Significant differences: * *p* < 0.05, *** *p* < 0.001.

The results of the correspondence of the manual delineation between two operators are presented in Figure 4. It is clearly visible that the histological images are stretched in the mediolateral direction in comparison to in vivo MR images (Figure 4A). The width-to-height ratio of the rat brain in the cross-sectional slice through the CC is 1.8 ÷ 1.9 vs. 2.1 ÷ 2.2 if the same measurement is completed in the matching histology slice. The CC in LFB-stained slice has more detectable bounds in comparison with the DAPI-stained image (Figure 4A). It was found that the similarity between CC delineation by two operators was significantly higher for LFB-stained sections in comparison with DAPI stained sections whereas similarities of CC delineation for MR images were similar (Figure 4B–D). Correspondence between operators was significantly higher for the brain section comparison with CC for all types of source images (Figure 4E). No differences were found between delineation of post-ischemic and sham-operated animals in both datasets and both delineated ROIs (Figure 4C).

### 3.2. Correspondence of Transformed and Manually Delineated ROIs

**Figure 5:**
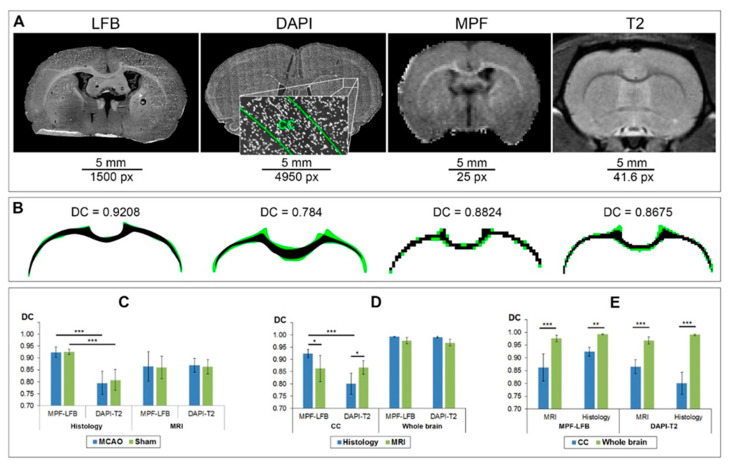
Correspondence between the transformed and delineated CC and brain section. (**A**) Example of ROI transformation of MR image to histological image. (**B**) Example of the ROI transformation of histological image to MR image. (**C**,**D**) Correspondence of the transformed and manually delineated CC and brain section in comparison with the correspondence between operators for CC (**C**) and brain section (**E**). Error bars correspond to the SD. Significant differences: * *p* < 0.05, ** *p* < 0.01, *** *p* < 0.001.

Figure 5 demonstrates the usage and validation of the ROIT method for MRI-to-histology (Figure 4A) as well as for histology-to-MRI ROI transformations (Figure 3B). Validation of the ROIT method included the comparison between manually delineated and transformed CC and brain section, as well as the comparison between application of ROI transformation and the usage of another operator (Figure 4C,D). The ROIT method showed excellent correspondence (DC > 0.9) for all datasets, types of image (MRI and histology), and types of ROI (CC and brain section) except DC for one of operators in CC ROI transformation from DAPI-stained image to T2 image (DC = 0.84 ± 0.02). For the sharply bounded brain section, manual delineation showed a significantly better correspondence between operators whereas for CC with less sharp edges the ROIT method demonstrated a significantly better correspondence or no differences (Figure 4C,D). The most expressed differences between the ROIT method and the manual delineation by two operators for the CC on DAPI-stained images were found (DC was 0.90 and 0.92 for the ROIT method vs. 0.80 for manual delineation) (Figure 5C).

### 3.3. Comparison of Measurements Performed by the ROIT Method and Manual Delineation

**Figure 6:**
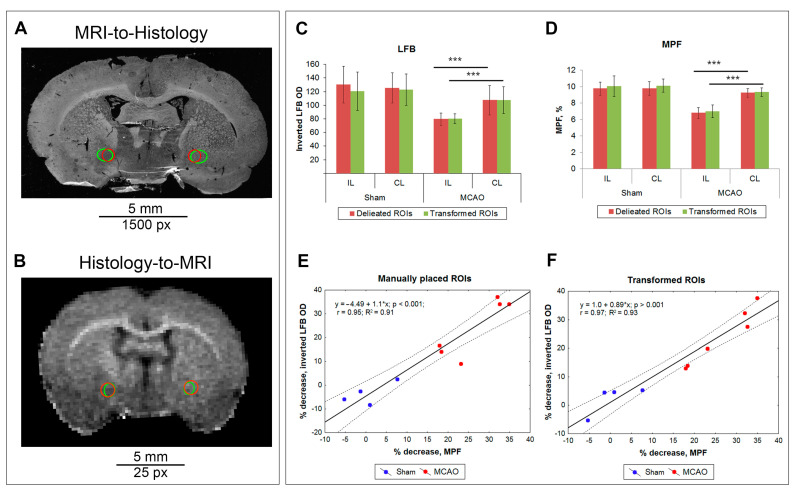
Correspondence of LFB OD and MPF measurements performed by the ROIT method and manual ROI placement on LFB-stained images and MPF maps in MCAO and sham-operated rats. (**A**,**B**) Example of the inverted red channel of the LFB image (**A**) and MPF map (**B**) of MCAO animal with ROIs into the ischemic lesion (IL) and contralateral side (CL). Manually placed round ROIs are marked by the red color; transformed ROIs are marked by the green color. (**C**,**D**) MPF (**D**) and LFB OD (**C**) measurements in the ischemic lesion (same size ROI in caudoputamen in sham-operated animals) and anatomically matched contralateral hemisphere. Error bars correspond to standard deviations. Significant differences: *** *p* < 0.001. (**E**,**F**) Linear regression plots of percentage changes in LFB OD, which were measured in manually placed (**E**) or transformed ROIs in the ischemic lesion relative to the symmetric contralateral anatomical region as a function of percentage changes in MPF.

To quantify the correspondence between the measurements using the ROIT method and the traditional manual ROI placement method, the LBF-MPF dataset was selected. For the percentage change in LFB OD and MPF in the ischemic lesion compared to the contralateral hemisphere, a significant correlation has previously been shown [9]. The results are presented in Figure 5. The figure shows that the manually placed (blue) and transformed (green) ROIs are in good agreement with each other for both histology (Figure 6A) and MRI (Figure 6B). At the same time, it is clearly visible that manually placed round ROIs were stretched in the horizontal direction during MRI-to-histology transformation and, conversely, compressed in the same direction during histology-to-MRI transformation. This corresponds to the direction of deformation of the histological image in comparison with MRI. The LFB and MPF measurements of the manually placed and transformed ROIs are almost identical and do not differ significantly for both post-ischemic and sham-operated animals (Figure 6C,D). After MCAO, a significant decrease in LFP and MPF in the lesioned hemisphere compared to the contralateral one was observed both when using the traditional manual measurements and when using the ROIT method. For both methods, a highly significant correlation (r = 0.95 for manual ROI placement, r = 0.97 for ROIT method) between the percentage change in LFP and MPF in the ischemic focus compared to the contralateral hemisphere was observed (Figure 6E,F).

## 4. Discussion

### 4.1. Validation of the ROIT Method

Validation of the ROIT method on the example of the CC and a brain section outline showed a high accuracy of ROI transformation in both directions, MR-to-histology, and histology-to-MRI. Method accuracy was demonstrated in two datasets. In the MPF-LFB dataset, when CC is clearly distinguishable on MR and histological images, the correspondence between the transformed and manually delineated ROIs was very high (DC was equal or exceeded 0.9 for both operators). It is important to note that for this dataset, the similarity of the source and transformed ROIs was not significantly different than the similarity of manual delineation between the operators. In more complicated cases, when the delineated CC was clearly visible on the MR image but poorly visible on the histological image, the differences in delineation between the operators were higher (DC = 0.80 ± 0.04); however, the ROIT method gave a better match in the results for both operators (DC = 0.90 ± 0.01 for OP1; DC = 0.92 ± 0.02 for OP2). In the case when the borders of the CC were less clear (transformation of ROI from a histological image stained with DAPI to a T2 image), the result of the transformation and delineation of CC boundaries were very different for the two operators. This highlights the importance of clearly defining the source ROI boundaries for the successful application of the ROIT method. We would like to emphasize the high accuracy of the ROIT method application for the deformed sections of post-ischemic animals: the DC for MCAO-operated animals did not differ from this indicator for sham-operated animals.

In addition, the feasibility of the method was demonstrated in terms of the consistency between the quantitative MRI and histological measurements: the consistency of MPF and LBF OD measurements did not differ between the transformed and manually placed regions. Moreover, the highly significant correlation between MPF and LFB OD measurements on the MCAO model, previously shown in our studies [9], was confirmed when using the manual method (r = 0.95, R^2^ = 0.91), and in the case of using the ROIT method, it turned out to be even higher (r = 0.97, R^2^ = 0.93).

### 4.2. Troubleshooting, Advantages, and Limitations of ROIT Method Application

#### 4.2.1. Troubleshooting

To ensure the successful application of the ROIT method, three critical points should be considered. First, an exact anatomical match between the MR and histological images is essential. Poor anatomical matching of the images makes all subsequent steps ineffective.

The second point of the successful registration is the clear identification of boundaries of at least several brain structures, such as the corpus callosum, lateral ventricles, intravenous capsule, commissures, etc., especially on the source image. We also found a lower accuracy of region transformation when DAPI staining was used as a source image. In this case, the qualification of the operator is of particular importance for proper image transformation.

Third, it is important to carefully choose landmarks for registration with the Big Warp tool of the ImageJ software. We advise using no more than 30 landmarks (20 on average) for registration using the most clearly identifiable points such as structure intersections, corners, and intact slice edges. It is important to check the similar location of landmarks in the superior–inferior direction since the histological section is usually stretched in the lateral direction.

#### 4.2.2. Advantages

The main advantage of the ROIT method is its applicability in solving complicated cases, for example, when it is impossible to determine the boundaries of the ROI on the histological image and there is no initial information about the measured parameter within this region. Specific examples may include the assessment of cell death, inflammation, regeneration, and in animal studies of stroke [8,45,46,47].

The ROIT method was successfully applied for the quantification of inflammation in an ischemic lesion [33]. The boundaries of the ischemic lesion were clearly visible on T2-weighted images. Activated microglia and macrophages can occupy a larger area in the immunostained images but can be distributed unevenly within the lesion; therefore, the boundaries of the lesion on the histological image cannot be clearly identified. Another example is quantifying the change in the MR signal after the genetic modification of brain cells [33,34,35] or the introduction of superparamagnetic particles [22,23,24,25,26,27,28,29,30,31,32], which can be clearly identified on histological images, while the localization of these cells or particles on the MRI image is not obvious.

Another example is quantifying the change in the MR signal after the genetic modification of brain cells or the introduction of paramagnetic particles, which can be clearly identified on histological images, while the localization of these cells or particles on the MRI image is not obvious. Thus, the ROIT method was applied in our recent study to quantify the change in the MR signal on T2*-weighted images after the intracerebral administration of genetic vectors that cause ferritin overexpression [33].

Finally, the method has the potential to be applied to compare MRI and human histological images, such as in a prostatectomy, although we have not tested it in this capacity.

Another advantage of the method is the reduction in subjectivity when choosing a ROI for measurements on the target image, corresponding to the ROI on the source image. Often the operator tends to take matching measurements exactly where they are clearly visible, even if they do not match well anatomically.

#### 4.2.3. Limitations

The developed ROIT method has several limitations. First, the size of the source ROIs to be transformed must not be too small relative to the size of the source MR image in pixels. Otherwise, even a slight displacement of the ROI during the transformation will lead to incorrect measurements. Secondly, as noted, the ROIT method is indispensable if the boundaries of the ROI for measurements in the source image are clearly identified, but there is no information about these boundaries in the target image. If the boundaries of the ROI are clearly visible in both images (for example, a specific brain structure with sharp boundaries), the ROIT method does not offer much advantage over manual delineation. Third, the ROIT method will not work correctly if the planes of the MR and histological slices are oblique. Fourth, the ROIT method cannot be applied if the images have local imperfections (noise, staining artifacts, discontinuities, etc.), which makes measurements in anatomically matched areas impossible, and ROIs should be slightly shifted. In this case, the manual placement of ROIs remains the only possible measurement method. Finally, the correctness of measurements when using the ROIT method significantly depends on the qualifications and experience of the operator, his knowledge of brain anatomy, and an expert assessment of the success of registering and transforming the region.

## 5. Conclusions

We have developed a simple region of interest transformation (ROIT) method based on existing Fiji (ImageJ) software tools for the anatomical matching of measurements on MRI and histological images. The ROIT plugin was developed for ImageJ to enable an automatization of the image processing and structural analysis of the rodent brain. The ROIT method solves the problems of matching measurements between MRI and histology images. The method can be used for the MRI-to-histology enlargement/transformation of ROI as well as for the histology-to-MRI ROI’s diminishment approach. The ROIT method was validated on two datasets and showed a high accuracy of ROI transformation both from an MR to a histology image, and a histology to MR transformation. The developed method is especially valuable for studies in which the boundaries of the ROI for measurements on one of the images (MR or histology) cannot be clearly determined.

## Data Availability

Data is contained within the article and supplementary material.

## Supplementary Materials

The following supporting information can be downloaded at: https://www.mdpi.com/article/10.3390/biomedicines10071556/s1, Video S1: The ROIT method implementation; Jar S1: plugin (https://drive.google.com/file/d/1BE_S4zaslwfA4nzgx5xjhITbQrWU20Gs/view, small program, utility) for automating the use of the method described in our article.
